# Cross-Modal Interaction Between Auditory and Visual Input Impacts Memory Retrieval

**DOI:** 10.3389/fnins.2021.661477

**Published:** 2021-07-26

**Authors:** Viorica Marian, Sayuri Hayakawa, Scott R. Schroeder

**Affiliations:** ^1^Department of Communication Sciences and Disorders, Northwestern University, Evanston, IL, United States; ^2^Department of Speech-Language-Hearing Sciences, Hofstra University, Hempstead, NY, United States

**Keywords:** multisensory integration, cross-modal interaction, audio-visual processing, auditory experience, visual memory, spatial memory, spoken words, environmental sounds

## Abstract

How we perceive and learn about our environment is influenced by our prior experiences and existing representations of the world. Top-down cognitive processes, such as attention and expectations, can alter how we process sensory stimuli, both within a modality (e.g., effects of auditory experience on auditory perception), as well as across modalities (e.g., effects of visual feedback on sound localization). Here, we demonstrate that experience with different types of auditory input (spoken words vs. environmental sounds) modulates how humans remember concurrently-presented visual objects. Participants viewed a series of line drawings (e.g., picture of a cat) displayed in one of four quadrants while listening to a word or sound that was congruent (e.g., “cat” or <meow>), incongruent (e.g., “motorcycle” or <vroom–vroom>), or neutral (e.g., a meaningless pseudoword or a tonal beep) relative to the picture. Following the encoding phase, participants were presented with the original drawings plus new drawings and asked to indicate whether each one was “old” or “new.” If a drawing was designated as “old,” participants then reported where it had been displayed. We find that words and sounds both elicit more accurate memory for *what* objects were previously seen, but only congruent environmental sounds enhance memory for *where* objects were positioned – this, despite the fact that the auditory stimuli were not meaningful spatial cues of the objects’ locations on the screen. Given that during real-world listening conditions, environmental sounds, but not words, reliably originate from the location of their referents, listening to sounds may attune the visual dorsal pathway to facilitate attention and memory for objects’ locations. We propose that audio-visual associations in the environment and in our previous experience jointly contribute to visual memory, strengthening visual memory through exposure to auditory input.

## Introduction

Many of us have had the experience of feeling transported back in time upon exposure to a familiar sight or sound – a song on the radio might conjure the image of your first car, or the sight of a tuba might invoke the cacophony of your middle school band. Such phenomena illustrate the essentially multisensory quality of what we experience, and subsequently, what we remember. While it is simple enough to intuit that memories made in one sensory modality could become associated with those in another by virtue of their shared context or source, here we ask whether the things we hear can directly alter how we encode and remember the things that we see.

Contrary to the traditional view of sensory processing as largely modality-specific and “bottom-up” in nature (e.g., from a sense organ up through modality-specific subcortical and cortical areas), there is now considerable evidence that dynamic networks of descending and lateral pathways enable higher-level functions (e.g., attention) to influence and optimize even very basic sensory processes (e.g., cochlear function in animals, Maison and Liberman, 2000; Darrow et al., 2006; Delano et al., 2007, and humans, e.g., Marian et al., 2018b), as well as the integration of inputs across modalities (most extensively researched with auditory and visual stimuli; Stein et al., 1996; Giard and Peronnet, 1999; Calvert et al., 2000; McDonald et al., 2000; Calvert, 2001; Molholm et al., 2002, 2004; Schroeder and Foxe, 2005; Driver and Noesselt, 2008; Talsma et al., 2010; Moran et al., 2013; Ten Oever et al., 2016). This work has contributed to our current understanding of sensory perception as an on-going interplay between stimulus-driven processing and top-down influence, both of which are characterized by significant cross-modal interactivity. Relatively less is known, however, regarding the nature of cross-modal interactivity and the role of perceptual experience in memory. The present study was therefore designed to examine the joint impact of multisensory exposure and experience on visual and spatial memory.

### Audio-Visual Interactions in Visual Perception

Robust behavioral evidence confirms that a range of visual processes, including detection, identification, and localization can be facilitated by the concurrent processing of auditory stimuli (Stein et al., 1996; Vroomen and De Gelder, 2000; Spivey et al., 2001; Molholm et al., 2004; Seitz et al., 2006; Van der Burg et al., 2008; Lupyan and Spivey, 2010; Salverda and Altmann, 2011; see also Shams et al., 2000; Morein-Zamir et al., 2003 for examples of visual distortion by auditory stimuli). There remains, however, considerable ambiguity and debate regarding the mechanisms underlying cross-modal facilitation, including the relative contributions of stimulus-driven bottom-up processing vs. top-down control (see De Meo et al., 2015 for discussion).

Cross-modal interactions of sensory information can occur at multiple stages of processing. Most commonly, we think of audio-visual integration as occurring in higher-level multisensory areas (e.g., superior temporal sulcus, middle temporal gyrus, inferior parietal cortex) where auditory and visual information about behaviorally relevant stimuli (e.g., objects and speech) can be brought together from separate processing streams to be integrated into a coherent percept (e.g., Calvert et al., 2000; Raij et al., 2000; Calvert, 2001; Beauchamp et al., 2004a, b; Powers et al., 2012). However, integration can also occur at lower levels, including in regions traditionally thought to be unisensory processing areas (e.g., the primary visual cortex V1; Giard and Peronnet, 1999; Molholm et al., 2002; Schroeder and Foxe, 2005; Watkins et al., 2006; Murray et al., 2016 for review), as well as in subcortical regions (e.g., multisensory neurons in the superior colliculus, Miller and D’Esposito, 2005), which receive descending projections from modality-specific subregions and can play a key role in stimulus localization and the control of orienting behaviors (Wallace, 2004). Furthermore, cross-modal interaction can be initiated at multiple stages concurrently in response to different factors (e.g., stimulus characteristics, contextual factors, top-down influences), and can shape perception and behavior via a variety of distinct mechanisms (e.g., attentional orienting, multisensory integration, cross-modal influence; see De Meo et al., 2015; Ten Oever et al., 2016).

**FIGURE 1:**
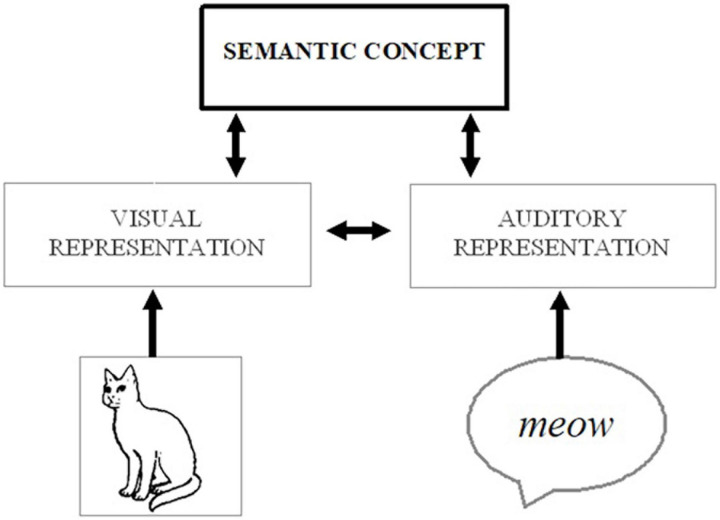
Possible processing route whereby activation from auditory (e.g., a meowing sound) or visual (e.g., a picture of a cat) stimuli could spread to a corresponding conceptual representation (e.g., of a cat) and associated visual features, thereby strengthening the salience of the visual object.

The mechanisms behind multisensory enhancement can be particularly ambiguous when either of the cross-modal inputs (on its own) would be sufficient to elicit a correct response (e.g., about an object’s location or identity; see Driver and Noesselt, 2008). For instance, it has been shown that people are better at identifying what object they are seeing (e.g., a cat) if they are provided with a redundant auditory cue (e.g., the sound of a cat, ¡meow¿; Chen and Spence, 2010). One explanation for this type of behavioral enhancement is that cross-modal interactions occur at early stages of processing, whereby exposure to the auditory stimulus automatically boosts attentional and perceptual processing of the visual input, strengthening the visual representation and facilitating identification. Alternatively, each signal could be processed up to and converge at the level of the stimulus meaning (visual image of a cat → visual representation of a cat → semantic concept of a cat ← auditory representation of <meow> ← auditory <meow>), and the dual activation of the semantic concept can make it easier to identify the image – either by engaging top-down processes to directly modulate perception or by affecting the decision or response criteria (without further involvement of perceptual/attentional processes; see Figure 1). Indeed, assuming the auditory input is a consistently reliable cue, it would be possible to correctly identify the “visual” target even if someone were to close their eyes, as the auditory stimulus would provide equally valid information about the object’s identity. In other words, while redundant signals could involve a high degree of cross-modal integration and feedback to affect modality-specific processing (e.g., boosting processing of the visual features), it could also be primarily bottom-up in nature and would not even necessarily require the integration of cross-modal information.

Now consider a case in which a person is asked to identify an object’s *location* from an array of pictures and is provided with the target ahead of time via a written word (e.g., cat). Assuming that was the only information the person received, the target word would be processed first (e.g., written word *cat* → orthographic representation of word *cat* → semantic concept of cat → visual features of a cat), which could then prepare the visual system to look for the corresponding visual object (e.g., by increasing sensitivity to the visual representation). In addition to top-down facilitation resulting from a previously presented target cue, visual search can be speeded if a congruent auditory sound (e.g., <meow>, or “cat”) is presented along with the visual display, even if it provides no information about the object’s location (Iordanescu et al., 2010; Lupyan and Spivey, 2010; Iordanescu et al., 2011). As with object identification, the auditory stimulus will begin with feed-forward activation, likely including associated visual features (e.g., auditory sound <meow> → auditory representation <meow> → concept of a cat → visual representation of a cat), which combined with the visual representation activated by the initial target word and the visual features in the display, can further boost activation and salience of the target object.

The key difference between these examples of object identification and localization is that in the latter case, the auditory cue is not sufficient to complete the task without any interaction with visual processing. In order to facilitate object localization, the auditory cue must improve how efficiently the *visual system* can take the final step of identifying the target’s location. In other words, because the auditory input does not itself provide information about the task-relevant external property (i.e., the location), we can conclude that if it facilitates localization, it must be doing so by directly modulating how the visual information is processed. Indeed, there is evidence that this kind of cross-modal interaction can occur at even more basic levels of processing, such as speeded detection of a visual target following a color change and a concurrent spatially uninformative sound (e.g., a beep, or “pip”; Van der Burg et al., 2008). In this case, the temporal congruence between the sound and a color change can boost visual attention at the critical time, helping to identify the location of the visual target. A burst of noise can also increase the perceived intensity of a concurrently-presented light in a different location (Stein et al., 1996), showing that auditory inputs can modulate the perceptual quality of visual stimuli. Lastly, hearing a sound can not only facilitate detection of a visual target appearing in the same place at the same time (Driver and Spence, 1998; Bolognini et al., 2005), but also when a visual target appears in a location *previously* cued by a sound (McDonald et al., 2000), providing strong evidence that auditory inputs can elicit top-down attentional orienting to a specific location, subsequently enhancing the perceptual salience of spatially coincident visual inputs.

### Audio-Visual Interactions in Visual Memory

While there is now substantial behavioral and neural evidence that cross-modal inputs can directly modulate attentional and perceptual processes, including perceptual learning of simple, artificial stimuli (e.g., dots and beeps; see Shams and Seitz, 2008 for review), relatively less is known about the influence of multisensory integration on memory for semantically meaningful, naturalistic objects. Given that multisensory integration has been shown to enhance the salience and attentional processing of sensory stimuli (Parkhurst et al., 2002; Santangelo and Spence, 2007; Matusz and Eimer, 2011; see Koelewijn et al., 2010; Talsma et al., 2010 for reviews), which can, in turn, strengthen memory encoding (Salthouse et al., 1984; Evans and Baddeley, 2018), it would be reasonable to expect that auditory facilitation of visual attention and perception could translate to better visual memory as well. Indeed, audio-visual encoding can enhance recognition memory for visual objects (Murray et al., 2004, 2005; Lehmann and Murray, 2005; Thelen et al., 2012, 2014, 2015; Moran et al., 2013; Thelen and Murray, 2013; Heikkilä et al., 2015; Matusz et al., 2015; Ueno et al., 2015; see Matusz et al., 2017 for review). For instance, in a series of experiments utilizing a continuous recognition task (identifying “old” vs. “new” pictures), Matusz et al. (2017) found that unimodal pictures (e.g., a cow) that were initially encoded along with a task irrelevant, but semantically congruent characteristic sound (e.g., “moo”) were later recognized with greater accuracy than unimodal stimuli or images paired with incongruent (e.g., “meow”) or neutral sounds (e.g., a tone).

Meyerhoff and Huff (2016) similarly found that recognition of semantically congruent audio-visual film clips was greater than of incongruent audio-visual clips. Unlike Matusz et al. (2017), however, memory for both congruent and incongruent audio-visual clips exceeded that of unimodal clips. The authors speculate that the relatively greater advantage for congruent audio-visual stimuli may stem from the multisensory integration of auditory and visual information in memory, resulting in more elaborate representations (analogous to perceptual advantages observed for congruent audio-visual stimuli; e.g., Chen and Spence, 2010). However, unlike perceptual integration, which typically requires precise temporal correspondence, audio-visual facilitation in memory was found to persist despite temporal asynchrony, leading the authors to conclude that cross-modal effects on memory are unlikely to be mere extensions of perceptual processes.

Advantages of multisensory encoding have also been extended to motion. Recognition memory is superior for moving relative to static images (Matthews et al., 2007). Similar to Matthews et al. (2007), Meyerhoff and Huff (2016) propose that the superior memory for moving pictures may result from the construction and storage of a scene-based “object file” (Hollingworth and Henderson, 2002) in long-term memory, which contains detailed representations of not only the visual forms of particular objects, but also their spatial positions within a larger scene. Hollingworth and Henderson (2002) theorized that visual fixations to different components of a scene play a key role in the formation of object files and that directing visual fixations and attention to the spatial locations of previously seen objects can facilitate visual memory retrieval. Matthews et al. (2007) build upon this model by proposing that visual objects in a scene are encoded along with not only spatial information, but temporal information as well, and that the activation of “motion schemata” facilitates subsequent recall of associated visuospatial memory traces.

These findings demonstrate that, like perceptual and attentional processes, memory can be influenced by exposure to multisensory stimuli. Unlike effects on perception, however, which have been documented using a wide variety of tasks (e.g., discrimination, localization, and detection), much of the existing work on multisensory memory has been limited to object recognition. Analogous to perceptual effects emerging from exposure to redundant audio-visual cues, enhanced visual recognition following congruent audio-visual encoding could result from the availability of two valid sources of information regarding an object’s identity (i.e., the visual and auditory memory trace) rather than better memory of the visual percept itself. In addition to potentially providing additional retrieval cues (if both auditory and visual inputs are presented at test), exposure to one cue during retrieval could initiate the rapid reactivation of the other, both of which could be used to recall the identity of a previously seen object (e.g., the *redintegration hypothesis of memory retrieval*; Nyberg et al., 2000; Wheeler et al., 2000; Moran et al., 2013).

What has yet to be determined is whether audio-visual interactions and top-down attentional allocation to visual inputs at the encoding stage could facilitate memory for an object’s features and visual context – in other words, whether hearing an auditory stimulus can influence how well the unimodal visual representation itself is remembered. The present study investigates this possibility by examining whether hearing a spatially uninformative auditory cue (i.e., a sound that does not correspond to a visual object’s position on the screen) can improve visual memory for an object’s *location*. If, as predicted, memory for an object’s location is enhanced by listening to a corresponding sound, this facilitation is unlikely to result from the rapid retrieval of the spatially invalid auditory memory trace. Instead, it would suggest that the visual memory trace itself is strengthened by audio-visual encoding, providing compelling evidence of cross-modal interactivity in sensory memory.

### The Role of Experience in Audio-Visual Interactions

In addition to examining how cross-modal inputs interact in memory, a second goal of the present research is to determine whether the impact of auditory stimuli on visual memory varies as a function of prior experience with particular types of sounds.

Audio-visual integration has long been known to be moderated by physical properties of stimuli in the environment, most notably spatial and temporal contiguity (with greater integration for inputs that are closer in space and time; Meredith and Stein, 1986; Frens et al., 1995; Royal et al., 2009), as well as by characteristics such as the stimuli’s motion relative to the observer (e.g., greater integration for those that are looming than receding; Cappe et al., 2009). These principles are ecologically sensible given that real-world stimuli originating from the same source are likely to correspond spatially and temporally, and approaching stimuli can pose a potential danger (e.g., a predator or a car), making efficient processing especially consequential. Similarly, the semantic meanings attached to sensory stimuli in real-world contexts can provide information about the probability that multiple inputs represent features of the same object (e.g., the sight and sound of a firetruck), and may be especially likely to reveal experience-dependent differences in cross-modal interactivity (e.g., between particular types of auditory and visual input). Due to repeated experience associating visual and auditory features of objects (e.g., seeing a cat while hearing “meow”), object-based processing of auditory stimuli may boost activation of the corresponding visual representation, thereby increasing its salience through top-down and/or lateral feedback mechanisms (see Iordanescu et al., 2010, 2011). This experience-based explanation is consistent with Iordanescu et al.’s (2011) finding that cross-modal facilitation occurs between commonly co-occurring forms of object-based stimuli (e.g., visual features and sounds of objects as we interact with them, visual features and vocalized labels, such as when naming objects; and written and vocalized labels, such as when reading aloud), but not between stimuli that are not commonly processed together during real-world experiences (written labels and characteristic sounds). In other words, audio-visual interactivity during sensory processing varies as a function of our prior experiences with specific combinations of auditory and visual stimuli.

Prior experience with particular forms of auditory input (e.g., characteristic sounds vs. linguistic labels) can additionally modulate the types of representations that are brought to mind, which can subsequently impact performance on visual tasks. For instance, Lupyan and Thompson-Schill (2012) propose that the concepts activated by words tend to be more categorical and prototypical than those activated by characteristic sounds. In a series of studies, the authors observed that when participants were instructed to indicate whether a picture was congruent or incongruent with an auditory cue, performance was enhanced when the visual objects were cued by a verbal label (e.g., “cat”) relative to a characteristic sound (e.g., a meowing sound; Experiments 1A–C). Importantly, this advantage for spoken words was greater for pictures that were rated as more “typical” representations of their referents, providing support for the hypothesis that words tend to activate prototypical exemplars. Furthermore, it was found that the label advantage was specific to nouns (e.g., the word “cat”), and was not found for verbs (e.g., the word “meowing”) or verbalized sound imitations (e.g., the word “meow”; Experiment 2). Together, these findings provide support for the notion that linguistic labels are distinct from characteristic sounds in that they are more likely to represent an abstract concept that encompasses any number of individual exemplars (e.g., the general concept of a dog), whereas a characteristic sound (e.g., of barking) is likely to invoke a more specific referent (e.g., a particular type of dog; see Waxman and Gelman, 2009). As a result, the concepts that are brought to mind in response to a linguistic label should be less idiosyncratic relative to characteristic sounds, which may subsequently facilitate the initial recognition of any given visual depiction.

The goal of the present study is twofold. First, we examine whether cross-modal facilitation observed during perceptual and attentional tasks extends to subsequent memory. Second, we examine whether effects of auditory input on visual memory vary as a function of prior experience with particular types of sounds. Specifically, we examine the possibility that the more concrete and exemplar-specific nature of characteristic sounds may, in some cases, promote *better* memory compared to linguistic labels, such as when attempting to remember where objects were previously seen. According to Edmiston and Lupyan (2015), environmental sounds can be considered “motivated” cues, in that the qualities of the auditory stimuli convey meaningful information regarding the physical source, including where it is relative to the observer. Spoken words, in contrast, are “unmotivated” cues in that they do not provide information about the specific physical source – while there are certainly situations in which one hears an object’s label while looking at its referent (e.g., “that’s my cat”), the referent is rarely the *source* of spoken words and is very often entirely absent. Having learned over years of experience that one is likely to see the physical features associated with an auditory stimulus (e.g., a cat) upon orienting to the location of an environmental sound (e.g., <meow>), but not a word (e.g., “cat”), it is possible that sounds will be more effective at engaging attentional processes dedicated to visuospatial localization compared to words. We therefore investigate whether memory for the locations of visual objects may be greater when they are initially encoded along with an environmental sound compared to a spoken word, even when both auditory cues are spatially uninformative.

### The Present Study

The present study was designed to test the following hypotheses regarding the nature of audio-visual interactivity in visual object memory:

Hypothesis 1: Semantic congruence between auditory and visual inputs will facilitate visual memory.

Specifically, in addition to facilitating recognition (i.e., “what”) of visual objects (replicating earlier findings), we predict that spatially uninformative auditory input will improve memory for objects’ locations, providing evidence that auditory input can modulate visual memory in the absence of redundant cues.

Hypothesis 2: Cross-modal facilitation will vary as a function of experience-dependent associations between particular auditory inputs (spoken words vs. environmental sounds) and specific visuospatial dimensions (“what” vs. “where”).

While recognition memory is expected to be facilitated by both spoken words and environmental sounds (due to their informational relevance for identifying *what* objects were seen), spatial memory may be selectively enhanced by environmental sounds due to repeated experience associating the spatial locations of visual objects with environmental sounds, but not words.

## Materials and Methods

### Participants

Forty-three young adults (mean age = 21.9; *SD* = 3.2; 79% female) participated in the experiment^1^. Memory for visual objects associated with spoken words and environmental sounds were completed in two separate blocks, with the order of the blocks counterbalanced across subjects. All participants provided written consent and the research reported in this manuscript was approved by the University Institutional Review Board (STU00023477).

### Stimuli

Different sets of stimuli were used for the congruent, incongruent, and neutral trials of the two experimental blocks. The spoken word block included two types of neutral stimuli (a tone and a pseudoword), while the environmental sound block included one type of neutral stimulus (a tone). The inclusion of both pseudowords and tones as baselines in the spoken word condition enabled us to examine potential differences between neutral verbal and non-verbal cues within the same experimental context, while also allowing us to directly compare the effects of meaningful spoken words vs. environmental sounds relative to the same baseline (i.e., neutral tones). The procedures were identical for spoken words and environmental sounds.

During the encoding phase, participants were presented with either 64 (for spoken words) or 60 (for environmental sounds) black and white pictures of objects selected from the *International Picture Naming Project Database* (Szekely et al., 2004). All pictures were similar in saturation and line thickness and were displayed on a computer screen with 2,650 × 1,440 resolution, with participants seated 80 cm away from the screen. Labels representing each of the objects in the spoken word and environmental sound blocks were matched on English frequency (SUBTLEXUS; Brysbaert and New, 2009), concreteness, familiarity, and imageability (MRC Psycholinguistic Database; Coltheart, 1981) across the spoken word and environmental sound blocks, as well as across lists within each block (see Supplementary Tables 1–3 for details). Picture-word and picture-sound pairs spanned many different categories (e.g., animals, instruments, and food) and care was taken to ensure that there was a similar number of items per semantic category across lists within each block^2^.

Spoken words had a mean duration of 801.99 ms (*SD* = 134.04), ranged from 445.99 ms to 997.89 ms, and were recorded at 44,100 Hz by a female native English speaker. Environmental sounds had a duration of 1,000 ms. Neutral tones were 1,000 ms sine waveforms ranging from 250 to 1,750 Hz in 100 Hz increments in the spoken word condition and from 300 to 2,200 Hz in 100 Hz increments in the environmental sound condition. Different tones were utilized on each trial in order to mimic the structure of the congruent, incongruent, and neutral spoken word trials and the congruent and incongruent environmental sound trials, where auditory cues varied from trial to trial and between the spoken word and environmental sound conditions. In this way, each tone, word, or sound was paired with a single picture regardless of condition or trial type. Every trial additionally included audio-visual stimuli during encoding, but only unimodal visual stimuli during retrieval (see “Procedure” for additional detail regarding the retrieval phase). Prior research has shown that memory is enhanced when the context of retrieval matches that of encoding (i.e., context-dependent memory; see Smith and Vela, 2001 for review), as well as for odd events and items that are distinct with respect to the surrounding context or stimuli (i.e., a distinctiveness or isolation effect; von Restorff, 1933; Dunlosky et al., 2000; Hunt and Lamb, 2001). To ensure that the sensory contexts of encoding were equally probable and dissimilar to those of retrieval across the different trial types, neutral words and sounds were chosen as controls in lieu of unimodal visual stimuli (the latter of which could benefit from a match in encoding and retrieval contexts and their relative distinctiveness during encoding). All auditory cues were amplitude normalized and presented through headphones using monophonic sound reproduction, so as to make them spatially uninformative. In other words, while the auditory cues did contain spatial information and would be perceived to emanate from the center point of the display, the spatial location of the sound was fixed across all trials and did not provide meaningful information regarding the spatial location of the visual object on the screen. The sound level was fixed at two bars on an iMac desktop computer for all participants.

The visual display was divided into 9 equally sized grid spaces, following a 3 × 3 grid. On each trial of both word and sound blocks, a single picture was presented in one of four positions (top left corner, top right corner, bottom left corner, bottom right corner, with each critical position separated by an empty grid space). The location of pictures was randomized across trials with the constraint that pictures on consecutive trials never appeared in the same spatial location.

#### Spoken Words

Each picture in the spoken word block was presented concurrently with an auditory cue in one of four trial types (16 trials each): *congruent word* (i.e., the English label for the depicted object; e.g., a picture of a shoe + “shoe”), *incongruent word* (i.e., the English label for an object from a different semantic category; e.g., a picture of a guitar + “apple”; see Supplementary Table 4), *neutral word* (i.e., a meaningless pseudoword; e.g., a picture of a snake + “fenip”), or *neutral tone* (i.e., a meaningless beep; e.g., a picture of a candle + a tonal beep).

Five lists of 16 objects and one list of pseudowords were compiled to create the picture-auditory cue pairs (see Table 1). Pseudowords were taken from Bartolotti and Marian (2012), were constructed to follow English phonotactic rules and matched in length (*M* = 5.94 letters) to the real word stimuli used in the present experiment (*M* = 5.88 letters; *p* = 0.902). Each of the five object lists was used as the visual or auditory stimulus set in one of the trial types. To illustrate, a participant may see pictures from List 1 paired with words from List 1 (*congruent word*), pictures from List 2 paired with words from List 3 (*incongruent word*), pictures from List 4 paired with pseudowords (*neutral word*), and pictures from List 5 paired with a tonal beep (*neutral tone*). The lists were rotated across participants; each of the lists served in each position an equal number of times across participants. The 64 trials were presented in 16 four-trial runs. Each of the four trial types was presented in each run, with the order of trial types counterbalanced across runs (i.e., each run included a congruent word, incongruent word, neutral word, and neutral tone trial in a different order).

**TABLE 1:** List of the spoken word stimuli used in the present study.

**List 1**	**List 2**	**List 3**	**List 4**	**List 5**	**Pseudowords**
Apple	Belt	Bride	Anchor	Bra	Acrip
Cast	Book	Church	Button	Branch	Appint
Castle	Cowboy	Desk	Cloud	Chair	Bakloo
Cigarette	Finger	Giraffe	Doctor	Chimney	Eazoond
Dinosaur	Flag	Glue	Funnel	Dentist	Fenip
Doll	Grapes	Hat	Hammock	Diaper	Fummawp
Dress	Hamburger	Mask	Igloo	Eggplant	Ganteh
Ear	Leaf	Mountain	Kangaroo	Lamp	Glolay
Eel	Lemon	Mushroom	King	Llama	Iyork
Magnet	Medal	Orange	Puzzle	Map	Lateep
Pear	Microscope	Pipe	Shoe	Needle	Munbo
Pillow	Mop	Pirate	Thermos	Nun	Nepri
Sandwich	Penguin	Pizza	Tire	Plate	Peftoo
Stethoscope	Pyramid	Porcupine	Tomato	Present	Shundoe
Submarine	Skeleton	Refrigerator	Watermelon	Shrimp	Toymeen
Unicorn	Tractor	Vest	Wig	Spaghetti	Unyops

#### Environmental Sounds

Each picture in the environmental sound block was presented concurrently with an auditory cue in one of three trial types (20 trials each): *congruent sound* (i.e., an environmental sound corresponding to the depicted object; e.g., a picture of a dog + a sound made by a dog <woof–woof>), *incongruent sound* (i.e., an environmental sound corresponding to an object from a different semantic category; e.g., a picture of a trumpet + a sound made by a motorcycle <vroom–vroom>), or *neutral sound* (i.e., a meaningless beep; e.g., a picture of a helicopter + a tonal beep).

Four lists of 20 objects (unique from those used for spoken words) were compiled to create the picture-sound pairs (see Table 2). Each of the four lists was rotated across participants to serve as the visual or auditory stimulus set in one of the trial types. The 60 trials were presented in 20 three-trial runs, with each run including each of the three trial types in a counterbalanced order (congruent sound, incongruent sound, neutral sound).

**TABLE 2:** List of the environmental sound stimuli used in the present study.

**List 1**	**List 2**	**List 3**	**List 4**
Airplane	Astronaut	Accordion	Ambulance
Basketball	Bomb	Banjo	Baby
Bell	Can	Cannon	Bicycle
Boat	Elephant	Dog	Chainsaw
Camera	Gorilla	Dragon	Chicken
Cat	Harmonica	Drill	Clock
Donkey	Horse	Glasses	Cow
Door	Lawn mower	Harp	Cymbals
Drums	Lips	Heart	Dolphin
Frog	Matches	Lightning	Duck
Goat	Monkey	Lion	Flute
Gun	Motorcycle	Microphone	Guitar
Hippopotamus	Owl	Microwave	Hammer
Jackhammer	Piano	Pig	Hands
Nose	Radio	Shower	Helicopter
Printer	Rain	Snake	Rocket
Robot	Telephone	Stapler	Seal
Saxophone	Tornado	Swords	Taco
Toilet	Trumpet	Train	Violin
Xylophone	Typewriter	Whistle	Whip

### Procedure

**FIGURE 2:**
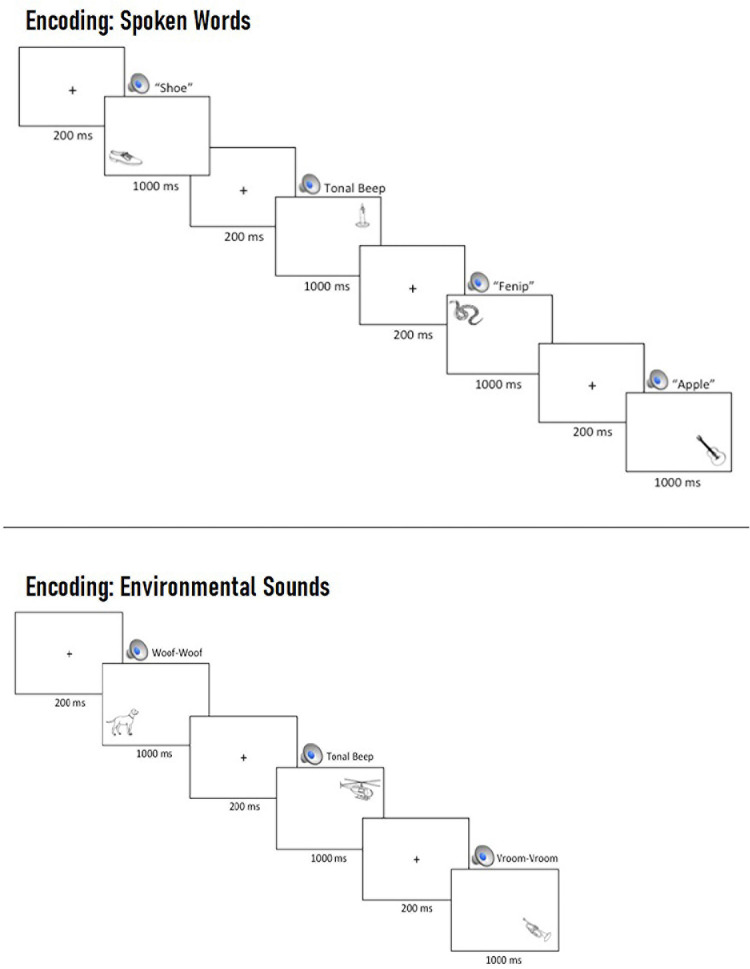
Example multi-trial run of spoken words **(Top)** and environmental sounds **(Bottom)** during encoding. On each trial, participants were presented with a central fixation cross for 200 ms, which was replaced by the concurrent presentation of a task-irrelevant, spatially uninformative auditory cue and a picture in one of four locations, which remained on screen for 1,000 ms prior to the beginning of the next trial.

Prior to beginning the task, participants were asked to remember the pictures for a later memory test while ignoring the auditory cues. At the beginning of each trial, a central fixation cross was presented for 200 ms, followed by the simultaneous presentation of an auditory cue and a picture, which remained on screen for 1,000 ms (see Figure 2).

After all encoding trials, participants completed a simple 5-min numerical filler task (determining which of two values is larger) before completing the retrieval task.

**FIGURE 3:**
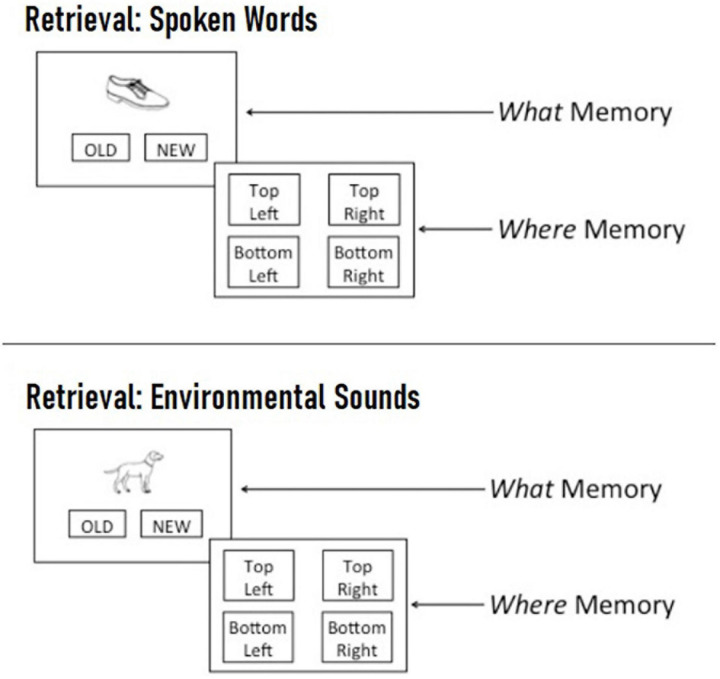
Example spoken word **(Top)** and environmental sound **(Bottom)** retrieval trial. On each trial, participants were first presented with a single picture in the center of the screen and asked to indicate whether they recognized it from the encoding phase by clicking “old” or whether it was not previously seen by clicking “new.” If a picture was designated as “old,” participants were then asked to indicate the spatial location where the picture appeared during the encoding phase by clicking on one of four quadrants labeled “top left,” “top right,” “bottom left,” or “bottom right.”

For the retrieval task, participants were presented with the 64 (spoken word) or 60 (environmental sound) pictures that appeared during the encoding task (i.e., “old” pictures), as well as an equal number of foil pictures that were not previously seen (i.e., “new” pictures). During the recognition phase of each trial, a single picture appeared in the center of the screen and participants were asked to click on *old* if the picture was previously seen during the encoding phase, and to click on *new* if it was not. If an image was designated as *old*, participants were asked to indicate which spatial location the picture appeared in during the encoding phase by clicking on one of four boxes located in the four corners of the screen (see Figure 3). Participants were instructed to respond as quickly as possible without sacrificing accuracy. Across the two blocks, participants completed a total of 124 encoding trials and 248 retrieval trials and the entire experiment lasted approximately 30 min.

### Data Analysis

We began with separate analyses of spoken words and environmental sounds, followed by a combined analysis. For both words and sounds, two separate generalized linear mixed-effects models were constructed to examine the effects of trial type (congruent, incongruent, neutral) on (1) recognition (“what”) and (2) location (“where”) accuracy for pictures that were previously seen during the encoding phase. Trial type was entered as a fixed effect and treatment coded to compare each level to congruent trials (i.e., congruent [0] vs. incongruent [1], neutral word [1], and neutral tone [1] trials for spoken words and congruent [0] vs. incongruent [1] and neutral tone [1] trials for environmental sounds). Models additionally included random intercepts for participant and target, as well as word frequency (zipf), concreteness, imageability, and familiarity of the targets’ labels as covariates.^3^ Following initial analyses comparing congruent trials to incongruent and neutral trials, planned pairwise comparisons were conducted to compare each of the incongruent and neutral trial types to each other. Follow-up analyses were additionally conducted on a subset of items (*N* = 35 out of 80), which were matched in semantic category across the spoken word and environmental sound lists (see Supplementary Table 4).

## Results

### Spoken Words

#### Recognition (“What”)

**FIGURE 4:**
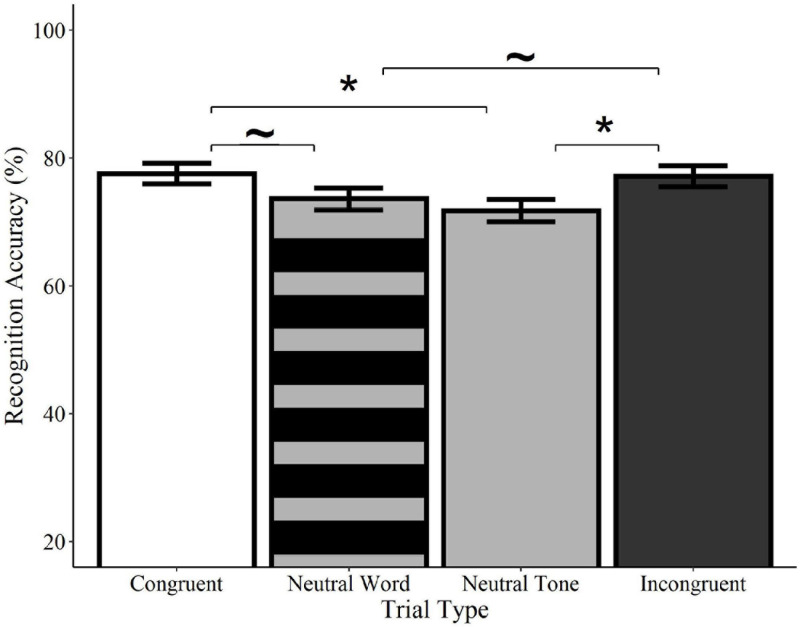
Effect of trial type on recognition accuracy for spoken words. Visual objects initially paired with congruent (white) or incongruent (black) spoken words were recognized with significantly greater accuracy than those paired with neutral tones (solid gray) and with marginally greater accuracy than pictures paired with neutral words (striped gray). Accuracy did not differ between neutral tone and neutral word trials or between congruent and incongruent trials. Error bars represent standard error. ^∼^*p* < 0.10, *p < 0.05.

Recognition accuracy was significantly higher on congruent trials relative to neutral tone trials (*Estimate* = −0.33, *SE* = 0.14, *z* = −2.41, *p* = 0.016) and marginally higher than neutral word trials (*Estimate* = −0.27, *SE* = 0.14, *z* = −1.94, *p* = 0.052; see Figure 4). Similarly, accuracy on incongruent trials was significantly higher than on neutral tone trials (*Estimate* = −0.30, *SE* = 0.14, *z* = −2.18, *p* = 0.029) and marginally higher than on neutral word trials (*Estimate* = −0.25, *SE* = 0.14, *z* = −1.77, *p* = 0.076). Accuracy did not differ between congruent and incongruent trials (*Estimate* = −0.4, *SE* = 0.14, *z* = −0.29, *p* = 0.769) or between neutral word and neutral tone trials (*Estimate* = −0.08, *SE* = 0.14, *z* = −0.55, *p* = 0.582)^4^. These findings indicate that memory for previously-seen objects is enhanced when they are paired with meaningful words, regardless of whether or not the words are congruent with the visual object. Concurrent presentation of meaningless non-words, on the other hand, resulted in accuracy scores that were numerically lower than those of meaningful words, but higher than meaningless tones.

Follow-up analyses on category-matched items revealed a similar pattern, with significantly higher recognition accuracy on congruent trials relative to both neutral tone (*Estimate* = −0.53, *SE* = 0.21, *z* = −2.48, *p* = 0.013) and neutral word trials (*Estimate* = −0.47, *SE* = 0.21, *z* = −2.19, *p* = 0.028) and marginally higher accuracy on incongruent trials relative to neutral tone (*Estimate* = −0.40, *SE* = 0.20, *z* = −1.93, *p* = 0.054) and neutral word trials (*Estimate* = −0.36, *SE* = 0.21, *z* = −1.71, *p* = 0.087). Congruent trials did not differ from incongruent trials (*Estimate* = −0.09, *SE* = 0.21, *z* = −0.40, *p* = 0.689) and neutral tone trials did not differ from neutral word trials (*Estimate* = −0.08, *SE* = 0.20, *z* = −0.42, *p* = 0.672).

#### Location (“Where”)

**FIGURE 5:**
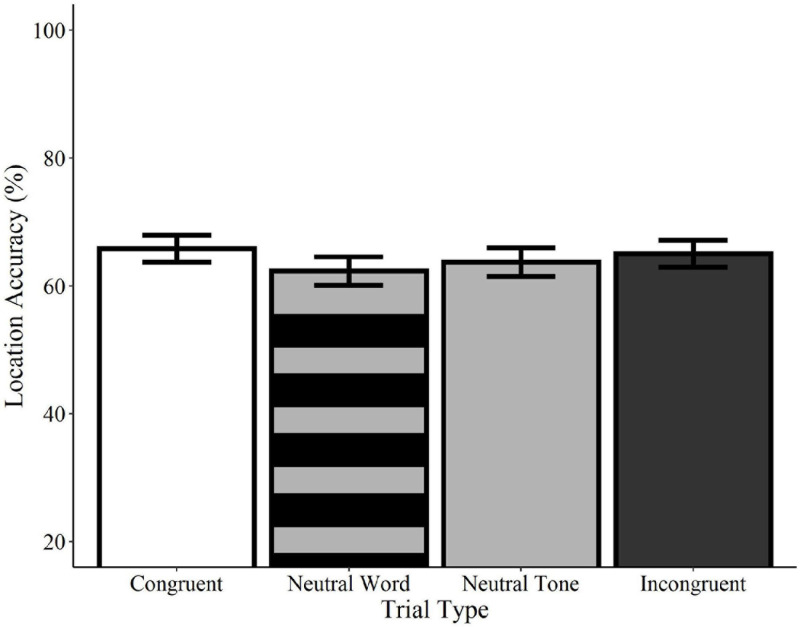
Effect of trial type on location accuracy for spoken words. Location accuracy did not differ between congruent (white), neutral word (striped gray), neutral tone (solid gray), and incongruent (black) spoken word trials. Error bars represent standard error.

Analyses of location accuracy revealed no significant differences between congruent trials and incongruent word, neutral word, or neutral tone trials (*ps* > 0.05; see Figure 5). Likewise, no differences were observed between incongruent trials and neutral word and tone trials or between neutral word and neutral tone trials (*ps* > 0.05). Similarly, no effects of trial type were found in any comparisons using the category-matched subset of items (*ps* > 0.05). Contrary to the effects observed for recognition memory, these results indicate that accuracy for the locations of visual objects is not influenced by the concurrent presentation of congruent or incongruent words relative to neutral words or tones.

### Environmental Sounds

#### Recognition (“What”)

**FIGURE 6:**
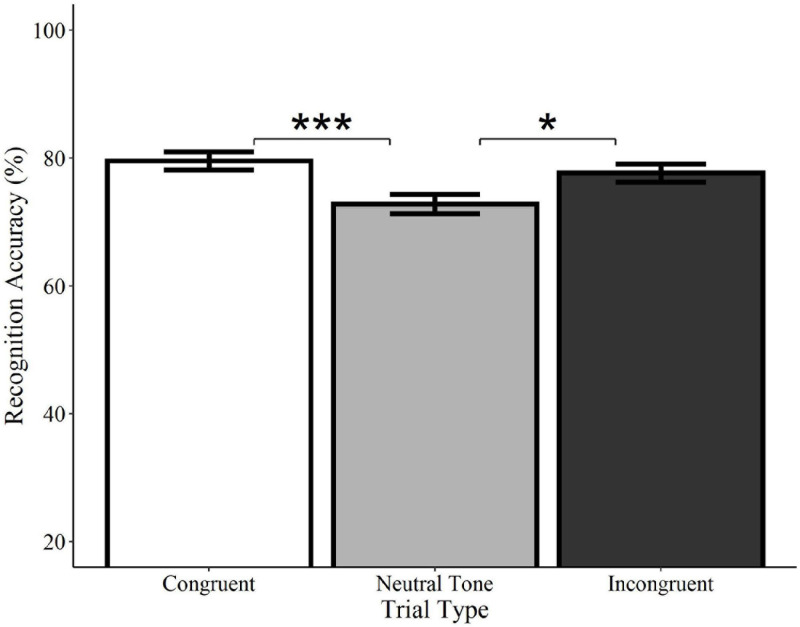
Effect of trial type on recognition accuracy for environmental sounds. Visual objects initially paired with congruent (white) or incongruent (black) environmental sounds were recognized with significantly greater accuracy than those paired with neutral sounds (gray). Congruent and incongruent trials did not significantly differ from each other. Error bars represent standard error. *p < 0.05, ***p < 0.001.

Recognition accuracy was significantly higher on congruent trials relative to neutral tone trials (*Estimate* = −0.41, *SE* = 0.12, *z* = −3.34, *p* < 0.001), as well as on incongruent trials relative to neutral tone trials (*Estimate* = −0.31, *SE* = 0.12, *z* = −2.46, *p* = 0.014; see Figure 6). Accuracy did not differ between congruent and incongruent trials (*Estimate* = −0.13, *SE* = 0.13, *z* = −0.99, *p* = 0.322)^5^. Consistent with the analyses of spoken words, these findings indicate that recognition for *what* objects were previously seen is enhanced by the concurrent presentation of meaningful sounds, regardless of whether they are semantically congruent with the visual object.

Follow-up analyses on category-matched items revealed a similar pattern, with significantly higher recognition accuracy on congruent trials relative to neutral tone trials (*Estimate* = −0.56, *SE* = 0.18, *z* = −3.06, *p* = 0.002) and on incongruent trials relative to neutral tone trials (*Estimate* = −0.45, *SE* = 0.19, z = 02.45, *p* = 0.014). Congruent trials did not differ from incongruent trials (*Estimate* = −0.12, *SE* = 0.19, *z* = −0.63, *p* = 0.530).

#### Location (“Where”)

**FIGURE 7:**
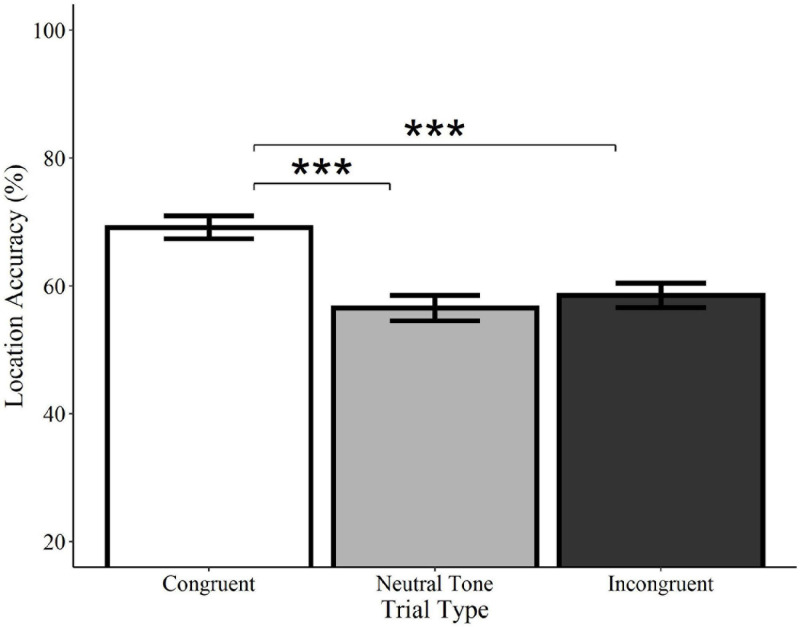
Effect of trial type on location accuracy for environmental sounds. Locations of visual objects initially paired with congruent (white) environmental sounds were remembered with significantly greater accuracy than those paired with neutral (gray) or incongruent (black) sounds. Neutral and incongruent trials did not significantly differ from each other. Error bars represent standard error. ***p < 0.001.

Location accuracy was significantly higher on congruent trials relative to both incongruent (*Estimate* = −0.54, *SE* = 0.12, *z* = −4.33, *p* < 0.0001) and neutral tone trials (*Estimate* = −0.58, *SE* = 0.13, *z* = −4.62, *p* < 0.0001; see Figure 7). Incongruent trials did not differ from neutral tone trials (*Estimate* = −0.04, *SE* = 0.12, *z* = −0.36, *p* = 0.722). Similarly, analyses of category-matched items revealed significantly higher location accuracy for congruent trials relative to incongruent (*Estimate* = −0.57, *SE* = 0.19, *z* = −.96, *p* = 0.003) and neutral tone trials (*Estimate* = −0.76, *SE* = 0.20, *z* = −3.85, *p* < 0.001) and no difference between incongruent and neutral tone trials (*Estimate* = −0.18, *SE* = 0.19, *z* = −0.93, *p* = 0.350). In other words, memory for objects’ locations is enhanced when they are initially encoded alongside a congruent, but not a neutral or incongruent sound, despite the fact that the sounds were not meaningful spatial cues.

### Comparison of Spoken Words and Environmental Sounds

Two additional models were constructed to directly compare the impact of spoken words and environmental sounds on recognition and location accuracy^6^. In each case, accuracy was entered as the outcome variable in a generalized linear mixed-effects model with fixed effects of trial type, input, and their interaction, plus random intercepts for participant and target. Both models included word frequency, concreteness, imageability, and familiarity of the targets’ labels as covariates. Trial type was treatment coded to compare congruent [0] to incongruent [1] and neutral (tone) trials [1] and input was treatment coded to compare words [0] to sounds [1]. A follow-up analysis compared incongruent trials [0] to neutral tone trials [1].

Recognition accuracy was significantly higher on congruent trials relative to neutral trials (*Estimate* = −0.33, *SE* = 0.14, *z* = −2.40, *p* = 0.017), as well as on incongruent trials relative to neutral trials (*Estimate* = −0.30, *SE* = 0.14, *z* = −2.18, *p* = 0.029). There was no main effect of input or interactions between input and any of the trial type contrasts (*ps* > 0.05).

For location accuracy, there was a significant interaction between input and the comparison of congruent to incongruent trials (*Estimate* = −0.48, *SE* = 0.18, *z* = −2.55, *p* = 0.011), as well as between input and the comparison of congruent to neutral trials (*Estimate* = −0.41, *SE* = 0.19, *z* = −2.15, *p* = 0.032; see above), confirming that congruent sounds, but not words, enhanced memory for object locations. No interaction was found between input and the comparison of incongruent trials to neutral trials (*Estimate* = 0.07, *SE* = 0.19, z = 0.37, *p* = 0.712) and there were no main effects of trial type or input (*ps* > 0.05).

## Discussion

The present experiment was designed to examine the extent and nature of audio-visual interactivity in visual memory, as well as the role of experience-dependent associations between cross-modal stimuli. Memory for objects’ identities (i.e., “what”) revealed that listening to meaningful spoken words or environmental sounds both enhanced memory for *what* visual objects were previously seen (relative to a neutral tone), regardless of whether or not the sounds were semantically congruent with the visual stimulus. One possibility is that the enhanced recognition memory in response to incongruent cues (relative to neutral cues) may be driven by expectancy violations, which have been shown to engage attentional processes (Parmentier et al., 2011; Vachon et al., 2012). Within the auditory modality, there is evidence to suggest that, under some conditions, environmental sounds may be recognized more accurately when embedded in an *incongruent* auditory context (e.g., the sound of cow mooing in a soundscape of a bowling alley) compared to a congruent auditory scene (e.g., sounds of a farm; Leech et al., 2009; Gygi and Shafiro, 2011). Gygi and Shafiro (2011) conjecture that such an Incongruency Advantage for recognition accuracy may arise from the relative novelty of incongruent stimuli and the sensitivity of sensory systems to contrasting events (Kluender et al., 2003; Ulanovsky et al., 2003). Similarly, Loftus and Mackworth (1978) found that participants studying images in preparation for a recognition memory task allocated greater visual attention to objects that were incongruent with the visual context (e.g., a picture of an octopus in a farm scene) than to objects that were contextually congruent (e.g., a picture of a tractor in a farm scene).

Alternatively (or additionally), the finding that both congruent and incongruent auditory inputs facilitated recognition memory relative to neutral tones may indicate that, regardless of the match between the visual and auditory objects, exposure to meaningful auditory stimuli initiates deeper or more elaborate semantic processing of the visual object that extends beyond its perceptual features (Craik and Lockhart, 1972; Craik and Tulving, 1975; Humphreys and Chalmers, 2016). For instance, Craik and Tulving (1975) found that recognition memory for previously seen words is enhanced following semantic judgments of the linguistic stimuli (e.g., category membership) compared to evaluations of their perceptual or phonological features (e.g., typescript and rhymes). Though participants in the present study were instructed to ignore the auditory stimuli, the enhanced memory for visual objects paired with meaningful words and sounds suggests that participants did engage in some level of auditory processing, and that meaningful auditory stimuli may promote more elaborate semantic processing of concurrently presented visual objects as well.

Importantly, however, there is reason to expect that basic semantic elaboration may have a more significant impact on memory for objects’ identities, which may be encoded semantically (e.g., “I saw a cat”; Wolfe, 1998; Konkle et al., 2010) and/or perceptually (e.g., the visual memory of a cat) than on memory for objects’ locations, which may rely more extensively on encoding of episodic perceptual details. Such an explanation could help account for the fact that recognition memory was facilitated by meaningful auditory inputs regardless of semantic congruency, while memory for *where* objects were previously seen was selectively enhanced by concurrent presentation of a semantically congruent, but not incongruent environmental sound or either congruent or incongruent spoken words. Marks (1989) found that semantic elaboration of pictures and their labels facilitated later recall of picture names, but not perceptual details. This finding is consistent with the view that visual objects can be encoded into memory via distinct semantic and perceptual (or episodic) pathways (e.g., dual-coding theory; Paivio, 1971, 1986), and that semantic elaboration may have a more robust impact on the former than the latter. There is additionally evidence that semantic dementia (Boxer et al., 2003; Davies et al., 2004; Moss et al., 2005; Patterson et al., 2007) and damage to areas that support semantic memory (e.g., subregions of the anterior temporal cortex; Bowles et al., 2007) disproportionately impact familiarity-based memory and object recognition relative to recollection-based episodic memory, which have been shown to be functionally and anatomically dissociable (see Brown and Aggleton, 2001; Eichenbaum et al., 2007; Ranganath and Ritchey, 2012 for reviews). For instance, Ranganath and Ritchey (2012) review evidence showing that the perirhinal cortex plays a substantial role in both familiarity-based recognition memory (e.g., for objects) and semantic processing, while the parahippocampal cortex is especially critical for the recollection of spatial and contextual details. To the extent that both congruent and incongruent auditory inputs can initiate deeper semantic processing of a concurrently presented visual object, exposure to either type of meaningful cue may subsequently facilitate recognition memory for the visual object’s identity.

In contrast, we theorize that the selective facilitation of spatial memory by congruent environmental sounds may primarily stem from effects of multisensory processing on *episodic* memory, and in particular, the formation of multisensory object-based representations (Kahneman et al., 1992) and their associated contexts (i.e., “event files”; Hommel, 2004). Eye-tracking studies have shown that when participants are prompted to recall information associated with a previously seen object, they will often make visual fixations to the object’s prior location, even if it is no longer visible (i.e., the “looking at nothing” phenomenon; Ferreira et al., 2008). This suggests that episodic visual memories are encoded along with spatial information, both of which may be reactivated during retrieval. Such effects have been observed both when participants are asked to recall features of the visual object itself (e.g., Umar et al., 2021), as well as when they are asked to recall auditory stimuli presented along with a visual stimulus (e.g., Hoover and Richardson, 2008). Critically, however, object-based encoding and attention appear to be highly contingent on causal relationships and spatiotemporal continuity among different features (Pylyshyn and Storm, 1988; Hoover and Richardson, 2008). For instance, Hoover and Richardson (2008) found that when participants were asked to recall semantic facts spoken to them by an animated rabbit, they made approximately equivalent numbers of fixations to the position where the rabbit had initially communicated the auditory information, as well as to a second location where the same rabbit reappeared after being shown to burrow underground between the two mounds. Importantly, participants did not preferentially fixate the second location when an identical rabbit appeared there from a different location off-screen, demonstrating that object-based binding of visual, auditory, and spatial inputs depends on real-world constraints. It may therefore be the case that object-based memory traces of visual objects and their spatial positions may be strengthened by the concurrent presentation of semantically congruent, but not incongruent sounds, particularly when the auditory cue is typically a reliable indicator of its referent’s physical location (i.e., environmental sounds, but not spoken words). Together, these findings suggest that hearing and seeing characteristics of the same object can facilitate visual memory, with the impact of auditory stimuli varying as a function of prior experiences with particular types of input.

### Audio-Visual Interactions in Visual Memory

There is now considerable evidence that even basic sensory processes can be impacted by cross-modal and top-down influences through lateral and descending pathways (see Macaluso and Driver, 2005; Driver and Noesselt, 2008 for reviews). For instance, Marian et al. (2018b) demonstrated that the brain can exert top-down control over the amplification of speech sounds in the cochlea, and does so selectively depending on whether complementary visual cues are available to aid in comprehension. Particularly relevant to the present investigation, prior work has demonstrated that spatially uninformative auditory cues (e.g., a tone) can increase attentional capture and the detection of visual targets (Vroomen and De Gelder, 2000; Van der Burg et al., 2008; Matusz and Eimer, 2011). For instance, Matusz and Eimer (2011) observed that detection of a visual target (e.g., a horizontal blue bar) was facilitated when a color-change cue in the same location was accompanied by a tone relative to when the visual cue was presented unimodally.

To date, however, the majority of studies investigating audio-visual interactivity has been restricted to cross-modal interactions during perceptual and attentional tasks. The present findings indicate that cross-modal interactions during the initial processing of complex, naturalistic objects modulate how visual information is subsequently remembered.

The finding that the concurrent presentation of meaningful auditory and visual cues enhances recognition of visual objects is consistent with prior work demonstrating facilitative effects of multisensory encoding (Murray et al., 2004, 2005; Lehmann and Murray, 2005; Thelen et al., 2012, 2014, 2015; Moran et al., 2013; Thelen and Murray, 2013; Heikkilä et al., 2015; Matusz et al., 2015, 2017; Ueno et al., 2015). Previously, this type of memory enhancement has often been attributed to cross-modal interactions during the *retrieval* of visual information from memory, such that re-exposure to a previously-seen visual object initiates rapid re-activation of corresponding perceptual experiences. For instance, prior work on multisensory memory has demonstrated that the retrieval of visual and auditory information is associated with similar neural activation patterns observed during the perception of multimodal stimuli (e.g., the visual and auditory cortex, respectively; Wheeler et al., 2000). Memory for *what* objects were previously seen can therefore benefit from two sources of relevant information – the auditory and visual memory traces pointing to the same object. While such an explanation implies a high degree of interactivity between auditory and visual representations stored in memory, it does not speak to the question of whether exposure to cross-modal sensory inputs changes how memories are encoded *within* a given modality (e.g., an effect of auditory input on visual memory). The results of the present study provide support for this possibility by showing that audio-visual processing can enhance memory for information encoded exclusively by the visual system. Given that the auditory inputs in the present study did not contain relevant information regarding where an object was previously seen, their facilitation of visuospatial memory suggests that cross-modal interactivity may modulate visual memory.

### The Role of Experience in Audio-Visual Interactions

In addition to demonstrating that auditory processing can enhance visual memory in the absence of redundant cues, the results of the present study suggest that the nature of cross-modal interactivity varies as a function of prior experience with particular forms of auditory and visual stimuli. Consistent with the observation that cross-modal facilitation is greater for combinations of audio-visual stimuli that commonly co-occur in naturalistic contexts (Iordanescu et al., 2011), the extent to which auditory stimuli can facilitate later memory for objects’ locations may depend on how reliably the spatial location of a given sound correlates with that of its associated referent during real-world experiences.

Characteristic sounds of objects are, by their very nature, physically tied to their source, and orienting to the location of an object’s sound is very likely to provide information about its visual properties. The source of spoken words, on the other hand, is often spatially displaced from that of its physical referent, making it an unreliable cue for the object’s location. Our discovery that environmental sounds are more likely to facilitate visual memory for object locations than spoken words (even when neither auditory cue is spatially informative) is consistent with the idea that the impact of auditory stimuli on visuospatial memory emerges as a result of experience-dependent changes to how the cognitive system responds to particular types of information. Furthermore, the present findings demonstrate that the processes engaged during real-world listening conditions (under which environmental sounds typically contain meaningful spatial information) persist even when auditory cues are presented monophonically and dissociated from the location of their visual referents.

One point to note, however, when interpreting the observed differences between spoken words and environmental sounds is that the two conditions differed in the proportion of congruent, incongruent, and neutral trials. Specifically, because the spoken word condition included two types of neutral trials (pseudowords and meaningless tones), congruent trials represented 1/4 of all spoken word trials, as compared to 1/3 of all environmental sound trials (which only included neutral tones). The greater advantage for spatial memory observed in response to congruent sounds compared to words could therefore be (at least in part) attributed to the fact that the congruent sounds constituted more reliable cues. The effects of trial type on *recognition* memory, however, were remarkably consistent across inputs, which speaks against this alternative explanation. To the extent that the higher proportion of neutral trials (relative to semantically congruent and incongruent trials) in the spoken word (vs. environmental sound) condition attenuated the semantic congruency effect on spatial memory, we would have expected to see a comparable reduction in the recognition memory advantage for meaningful (congruent/incongruent) vs. meaningless (neutral) cues. Instead, we observed that recognition memory was facilitated by meaningful words and sounds to a comparable degree relative to neutral cues. Furthermore, given that the ratio of congruent-to-incongruent trials was equivalent across input conditions, it is unlikely that a higher proportion of neutral trials would modulate the relative impact of congruent vs. incongruent cues. Nonetheless, while it is clear that congruent environmental sounds can facilitate memory for objects’ spatial locations, the relative advantage of congruent sounds over words should be confirmed in future studies using equal proportions of congruent, incongruent, and neutral trials across the two input conditions. Future studies would additionally benefit from assessing the rate and accuracy of identification for spoken word vs. environmental sound stimuli. For instance, our finding that spatial memory was enhanced in response to congruent environmental sounds (but not congruent words or neutral/incongruent sounds and words) could conceivably be attributed to greater identifiability of sound vs. word stimuli. Given that prior research indicates that environmental sounds are typically recognized at comparable, or even lower rates relative to spoken words (Uddin et al., 2018; Bartolotti et al., 2020), however, the advantage for congruent environmental sounds observed in the present study is unlikely to be attributable to greater recognition of sounds vs. words. We note that the identifiability of items within a given block (words and sounds) is unlikely to account for effects of condition (congruent, incongruent, and neutral), as lists were counterbalanced across participants so that each item was presented in each of the conditions (i.e., as the congruent and incongruent auditory stimulus, as well as the visual target).

Our findings reveal a close link between the sights and sounds of memory, evident in the enhancement of visuospatial memory by auditory experience – a finding consistent with the well-established impact of prior experience on perceptual processing. For instance, long-term experience with cognitively and perceptually demanding activities like music (e.g., Bidelman, 2016) and bilingualism (e.g., Marian et al., 2018a; Bidelman and Heath, 2019) can impact susceptibility to perceptual illusions. These include audio-visual illusions in which visual inputs bias the perceived location (e.g., the Ventriloquist Effect; Choe et al., 1975; see Vroomen and De Gelder, 2004) or identity (e.g., the McGurk Effect; McGurk and MacDonald, 1976; see Tiippana, 2014) of auditory input, as well as those characterizing effects of auditory signals on visual perception (e.g., the Double-Flash Illusion; Shams et al., 2000; see Keil, 2020). In fact, even short-term training with audio-visual stimuli can influence unimodal processing within the auditory system (e.g., Hazan et al., 2005; Song et al., 2008; Moradi et al., 2017), as well as the visual system (e.g., Eberhardt et al., 2014; Setti et al., 2014).

## Conclusion

In sum, our coherent perception of the world relies on the brain’s ability to continuously learn and predict relationships between cross-modal stimuli – those streaming in from the external environment, as well as those stored in memory based on prior experiences. Far from a modular view of the mind (Fodor, 1983), it is now clear that information derived from different modalities is used to guide even the most basic sensory processes (Churchland, 1988; Uttal, 2001; Prinz, 2006; Marian et al., 2018b; Spence, 2020). Experience with visual and auditory stimuli can have a bi-directional impact on perception and memory, where what we hear will influence what we see, what we see will influence what we hear, and what we perceive will contribute to our memory and mental models of the world. Consistent with this iterative view of cross-modal interaction, we find that listening to meaningful sounds can enhance memory for the identity and location of visual objects, and propose that visual memory may be influenced by bottom-up processing of audio-visual input, as well as top-down effects of audio-visual experience. We conclude that cross-modal interactivity in the cognitive architecture generates a cycle in which experience shapes memory and memory shapes experience.

## Data Availability Statement

The raw data supporting the conclusions of this article are available by the corresponding author upon request.

## Ethics Statement

The studies involving human participants were reviewed and approved by the University Institutional Review Board. The patients/participants provided their written informed consent to participate in this study.

## Author Contributions

VM was responsible for project administration and funding acquisition. SS and VM conceptualized and designed the experiments. SS and research assistants collected the data. SH and SS analyzed the data. SH drafted and revised the manuscript. VM and SS provided critical feedback on the manuscript. All authors contributed to the article and approved the submitted version.

## Conflict of Interest

The authors declare that the research was conducted in the absence of any commercial or financial relationships that could be construed as a potential conflict of interest.

## Publisher’s Note

All claims expressed in this article are solely those of the authors and do not necessarily represent those of their affiliated organizations, or those of the publisher, the editors and the reviewers. Any product that may be evaluated in this article, or claim that may be made by its manufacturer, is not guaranteed or endorsed by the publisher.

## Footnotes

1 Data from two participants who were included in environmental sound analyses were not analyzed for spoken words due to technical errors.

2 The number of items per category varied across blocks due to the constraint that objects in the environmental sound category needed to be associated with a recognizable sound. Follow-up analyses were therefore conducted on a subset of items that were matched in the number of items per category across the word and sound blocks (see Supplementary Table 4 for additional detail).

3 Models including covariates plus random slopes for trial type failed to converge. Similar patterns of results were obtained when models excluded covariates and included random intercepts for participant and target, as well as by-participant and by-target random slopes for trial type (i.e., the maximal random effects structure, Barr et al., 2013).

4 Similar patterns were observed when recognition accuracy scores for previously seen pictures were replaced with *d*′prime scores which additionally accounted for false alarms and correct rejections of new items. *D*′prime scores were higher for congruent trials relative to both neutral word [*Estimate* = −0.14, *SE* = 0.07, *t*(120) = −2.05, *p* = 0.042] and neutral tone trials [*Estimate* = −0.19, *SE* = 0.07, *t*(120) = −2.82, *p* = 0.006]. Likewise, *d*′prime scores were higher for incongruent trials relative to neutral word [*Estimate* = −0.12, *SE* = 0.06, *t*(40) = −2.09, *p* = 0.043] and neutral tone trials [*Estimate* = −0.17, *SE* = 0.07, *t*(40) = −2.53, *p* = 0.016]. Scores did not differ between congruent and incongruent trials or between neutral word and neutral tone trials (*ps* > 0.05).

5 Similar patterns were observed for *d*′prime scores, which were significantly higher for congruent trials relative to neutral tone trials [*Estimate* = −0.21, *SE* = 0.07, *t*(84) = −2.98, *p* = 0.004], as well as for incongruent trials relative to neutral tone trials [*Estimate* = −0.17, *SE* = 07, *t*(42) = −2.40, *p* = 0.021]. Scores did not differ between congruent and incongruent trials (*p* = 0.518).

6 Models comparing words to sounds included all congruent and incongruent trials, as well as the neutral tone trials. Neutral word trials exclusive to the spoken word task were excluded from the analyses.

## Supplementary Material

Supplementary Table 1 Mean word frequency, concreteness, familiarity and imageability of word stimulus lists. Word frequency (zipf) was calculated based on SUBTLEXUS (Brysbaert and New, 2009), and concreteness, familiarity, and imageability ratings were taken from the MRC Psycholinguistic Database (Coltheart, 1981). Numbers in parentheses represent standard deviations.

Supplementary Table 2 Mean word frequency, concreteness, familiarity and imageability of labels in sound stimulus lists.

Supplementary Table 3 Comparison of word frequency, concreteness, familiarity, and imageability of labels in word and sound lists.

Supplementary Table 4 Number of items per semantic category in the spoken word and environmental sound blocks in the main and follow-up (matched) analyses. Items in the follow-up matched analyses were chosen by including all items from the block containing the fewest objects per category (e.g., “ear,” “finger,” “skeleton” from *anatomy* in the spoken word block) and selecting a corresponding object from the more populated block that most closely resembled each object in the less populated block (e.g., “nose,” “lips,” “heart,” respectively in the environmental sound block).
