# Supplementary material for: Cross-Modal Interaction Between Auditory and Visual Input Impacts Memory Retrieval
Source: Front Neurosci. 2021 Jul 26;15:661477. doi: 10.3389/fnins.2021.661477 (PMC8350348; doi:10.3389/fnins.2021.661477)
Supplement: Supplementary Table 1 — Mean word frequency, concreteness, familiarity and imageability of word stimulus lists. Word frequency (zipf) was calculated based on SUBTLEXUS (Brysbaert and New, 2009), and concreteness, familiarity, and imageability ratings were taken from the MRC Psycholinguistic Database (Coltheart, 1981). Numbers in parentheses represent standard deviations. [file Table_1.docx]

**Supplemental Materials**

Table A1. Mean word frequency, concreteness, familiarity and imageability of word stimulus lists

|  | Word_1 | Word_2 | Word_3 | Word_4 | Word_5 | *p-value* |
| --- | --- | --- | --- | --- | --- | --- |
| Word Frequency | 3.96 (0.02) | 3.97 (0.02) | 4.08 (0.02) | 3.84 (0.02) | 3.99 (0.02) | 0.863 |
| Concreteness | 596.91 (1.94) | 598.33 (1.75) | 592.38 (1.64) | 578.8 (1.8) | 582.64 (1.9) | 0.559 |
| Familiarity | 511 (1.82) | 511.38 (1.82) | 524.91 (1.71) | 527.64 (1.72) | 549.17 (1.86) | 0.878 |
| Imageability | 583.55 (1.9) | 592.4 (1.83) | 602.1 (1.86) | 575 (1.87) | 581.64 (1.89) | 0.909 |

*Note.* Word frequency (zipf) was calculated based on SUBTLEXUS (Brysbaert & New, 2009), and concreteness, familiarity, and imageability ratings were taken from the MRC Psycholinguistic Database (Coltheart, 1981). Numbers in parentheses represent standard deviations.
