# Supplementary material for: Cross-Modal Interaction Between Auditory and Visual Input Impacts Memory Retrieval
Source: Front Neurosci. 2021 Jul 26;15:661477. doi: 10.3389/fnins.2021.661477 (PMC8350348; doi:10.3389/fnins.2021.661477)
Supplement: Supplementary Table 2 — Mean word frequency, concreteness, familiarity and imageability of labels in sound stimulus lists. [file Table_2.docx]

**Supplemental Materials**

Table A2. Mean word frequency, concreteness, familiarity and imageability of labels in sound stimulus lists

|  | Sound_1 | Sound_2 | Sound_3 | Sound_4 | *p-value* |
| --- | --- | --- | --- | --- | --- |
| Word Frequency | 4.09 (0.02) | 4.18 (0.02) | 4.11 (0.02) | 4.11 (0.02) | 0.983 |
| Concreteness | 619.09 (2.01) | 593.79 (2.18) | 589.08 (2.08) | 603 (2.04) | 0.103 |
| Familiarity | 519.83 (1.79) | 547.36 (2.01) | 502 (1.85) | 524.46 (1.85) | 0.639 |
| Imageability | 605.27 (1.96) | 598.71 (2.2) | 606 (2.14) | 610.46 (2.15) | 0.681 |

*Note.* Word frequency (zipf) was calculated based on SUBTLEXUS (Brysbaert & New, 2009), and concreteness, familiarity, and imageability ratings were taken from the MRC Psycholinguistic Database (Coltheart, 1981). Numbers in parentheses represent standard deviations.
