# Supplementary material for: Cross-Modal Interaction Between Auditory and Visual Input Impacts Memory Retrieval
Source: Front Neurosci. 2021 Jul 26;15:661477. doi: 10.3389/fnins.2021.661477 (PMC8350348; doi:10.3389/fnins.2021.661477)
Supplement: Supplementary Table 3 — Comparison of word frequency, concreteness, familiarity, and imageability of labels in word and sound lists. [file Table_3.docx]

**Supplemental Materials**

Table A3. Comparison of word frequency, concreteness, familiarity, and imageability of labels in word and sound lists

|  | Word | Sound | *p-value* |
| --- | --- | --- | --- |
| Word Frequency | 3.97 (0.59) | 4.12 (0.77) | 0.16 |
| Concreteness | 589.53 (44.86) | 600.34 (42.13) | 0.168 |
| Familiarity | 524.32 (65.21) | 523.53 (67.63) | 0.303 |
| Imageability | 586.55 (41.65) | 604.98 (39.42) | 0.103 |
