# Supplementary material for: Cross-Modal Interaction Between Auditory and Visual Input Impacts Memory Retrieval
Source: Front Neurosci. 2021 Jul 26;15:661477. doi: 10.3389/fnins.2021.661477 (PMC8350348; doi:10.3389/fnins.2021.661477)
Supplement: Supplementary Table 4 — Number of items per semantic category in the spoken word and environmental sound blocks in the main and follow-up (matched) analyses. Items in the follow-up matched analyses were chosen by including all items from the block containing the fewest objects per category (e.g., “ear,” “finger,” “skeleton” from anatomy in the spoken word block) and selecting a corresponding object from the more populated block that most closely resembled each object in the less populated block (e.g., “nose,” “lips,” “heart,” respectively in the environmental sound block). [file Table_4.docx]

**Supplemental Materials**

Table A4. Number of items per semantic category in the spoken word and environmental sound blocks in the main and follow-up (matched) analyses.

|  | | Word | | Sound | | Word (Matched) | | Sound (Matched) | |
| --- | --- | --- | --- | --- | --- | --- | --- | --- | --- |
| anatomy (e.g., *ear*) | 3 | | 4 | | 3 | | 3 | |  |
| animal (e.g., *lion*) | 9 | | 20 | | 9 | | 9 | |  |
| apparel (e.g., *dress*) | 11 | | 0 | | 0 | | 0 | |  |
| food (e.g., *sandwich*) | 14 | | 1 | | 1 | | 1 | |  |
| houseware (e.g., *plate*) | 12 | | 3 | | 3 | | 3 | |  |
| instrument (e.g., *piano*) | 0 | | 13 | | 0 | | 0 | |  |
| nature (e.g., *rain*) | 4 | | 3 | | 3 | | 3 | |  |
| person (e.g., *bride*) | 7 | | 2 | | 2 | | 2 | |  |
| recreation (e.g., *puzzle*) | 3 | | 2 | | 2 | | 2 | |  |
| building (e.g., *church*) | 5 | | 0 | | 0 | | 0 | |  |
| tool/device (e.g., *microwave*) | 10 | | 24 | | 10 | | 10 | |  |
| transport (e.g., *airplane*) | 2 | | 8 | | 2 | | 2 | |  |
| *Total* | 80 | | 80 | | 35 | | 35 | |  |

*Note.* Items in the follow-up matched analyses were chosen by including all items from the block containing the fewest objects per category (e.g., “ear,” “finger,” “skeleton” from *anatomy* in the spoken word block) and selecting a corresponding object from the more populated block that most closely resembled each object in the less populated block (e.g., “nose,” “lips,” “heart,” respectively in the environmental sound block).
